# Triglyceride-glucose index is associated with poor sleep quality in apparently healthy subjects: A cross-sectional study

**DOI:** 10.20945/2359-3997000000517

**Published:** 2022-09-20

**Authors:** Daniela Carolina Avelino, Alessandra da Silva, Larissa Oliveira Chaves, Júlia Cristina Cardoso Carraro, Fernanda de Carvalho Vidigal, Josefina Bressan

**Affiliations:** 1 Universidade Federal de Viçosa Departamento de Nutrição e Saúde Viçosa MG Brasil Departamento de Nutrição e Saúde, Universidade Federal de Viçosa, Viçosa, MG, Brasil; 2 Universidade Federal de Ouro Preto Departamento de Nutrição Clínica e Social Ouro Preto MG Brasil Departamento de Nutrição Clínica e Social, Universidade Federal de Ouro Preto, Ouro Preto, MG, Brasil; 3 Universidade Federal de Alfenas Faculdade de Nutrição Alfenas MG Brasil Faculdade de Nutrição, Universidade Federal de Alfenas, Alfenas, MG, Brasil

**Keywords:** Cardiometabolic risk, insulin resistance, ROC curve, sleep quality, TyG index

## Abstract

**Objectives:**

We aimed to evaluate the association between the triglyceride glucose index (TyG index) and sleep quality and to establish a cut-off value for the TyG index based on the prevalence of subjects with insulin resistance (IR).

**Materials and methods:**

This cross-sectional study involved Brazilian health professionals (20-59 years). A total of 138 subjects answered the Pittsburgh Sleep Quality questionnaire to evaluate sleep quality. They were categorized into two groups: good sleep quality (global score ≤ 5 points) and poor sleep quality (global score ≥ 6 points). Also, we classified the subjects as having a high (>8.08 or >4.38) or low TyG index (≤8.08 or ≤4.38).

**Results:**

The majority of the subjects (70%) with high TyG index values (>8.08 or >4.38) reported poor sleep quality (p ≤ 0.001). Those with poor sleep quality had a 1.44-fold higher prevalence of IR (TyG index >8.08 or >4.38) compared to those with good sleep quality, regardless of sex, total cholesterol, LDL/HDL ratio, insulin, complement C3, CRP, and adiponectin (p ≤ 0.001).

**Conclusion:**

Our data showed a positive and significant association between the TyG index and poor sleep quality. Thus, these findings support the association between poor sleep quality and IR.

## INTRODUCTION

Insulin resistance (IR) is a condition in which the molecular mechanisms of insulin uptake and degradation are impaired, leading to the development of type 2 diabetes (T2D) and cardiovascular diseases in the long term (1–3). More than 500 million individuals were living with T2D globally in 2018, and it is expected to have a high prevalence in low-income countries (4). Adults with diabetes have a higher risk for all-cause morbidity and mortality because they often present other major comorbidities such as cardiovascular, chronic lower respiratory, and kidney diseases (5). These complications are mediated by several inflammatory markers, including cytokines such as tumor necrosis factor-alpha (TNF-α), interleukin-1 beta (IL-1β), and interleukin-6 (IL-6), that trigger an inflammatory response (6–8). Since IR can contribute to the pathogenesis of diabetes and its related comorbidities (3,9,10), understanding its mechanisms is of great importance.

Poor sleep quality is a common issue in modern society for several reasons, and growing evidence has linked it with IR (11–14). For example, as a direct consequence of the COVID-19 pandemic, sleep problems have affected approximately 40% of people in general and healthcare populations (15–18). While short sleep duration and metabolic impairments are strongly associated (19), their mechanisms remain largely unknown. There is some support for the roles of the hypothalamic-pituitary-adrenal (HPA) axis and sympathetic activation in glucose impairments and IR due to inadequate sleep quality (20,21).

The triglyceride glucose index (TyG index) has been extensively used as a reliable marker for IR, expressed as the product of triglyceride and glucose levels (22,23). A meta-analysis showed a significant association between higher TyG values and T2D risk (24). Recently, two cross-sectional studies have associated TyG with obstructive sleep apnea (OSA), a sleep breathing disorder that often involves IR (25,26). However, the relationship between TyG and sleep quality has not been previously studied.

Given the limited evidence on the association between TyG index and sleep quality, we aimed to establish a cutoff value for the TyG index based on the prevalence of IR patients under the homeostatic model assessment of IR (HOMA-IR) and evaluate the association between TyG index and sleep quality. We hypothesize that higher TyG index values are positively associated with poor sleep quality.

## MATERIALS AND METHODS

This study followed the Strengthening the Reporting of Observational Studies in Epidemiology (STROBE) statement. The STROBE checklist used is included in the Supplementary Material.

### Study design and subjects

We analyzed data collected by a previous cross-sectional study in Viçosa, Brazil, that involved Brazilian health professionals between 20 and 59 years old (27). All subjects signed a consent form previously approved by the Human Research Ethics Committee of the Federal University of Viçosa (Ref. No. 005/2011; Viçosa, Brazil) under the principles of the Declaration of Helsinki. This study is not registered in “Plataforma Brasil” because it was approved on February 18, 2011, before “Plataforma Brasil” came into effect on January 2, 2012.

To be eligible for this study, health professionals (doctors, nurses, nutritionists, physical trainers, physiotherapists, dentists, pharmacists, biochemists, and psychologists) must work in health facilities or higher education institutions, and students must be in their last two years of courses in a health-related area. The recruitment was performed via phone calls, website disclosures, social networks, local radio, and pamphlets. Individuals who were pregnant, lactating, using corticosteroids, using antibiotics, had a cancer diagnosis within the last three years, or had any serious illness that required hospitalization at the time of this study, were excluded. Individuals who could not follow the measuring protocols such as weighing, blood pressure, or performing blood collection were also excluded. All data were collected between January 2012 and July 2013.

**Figure 1:**
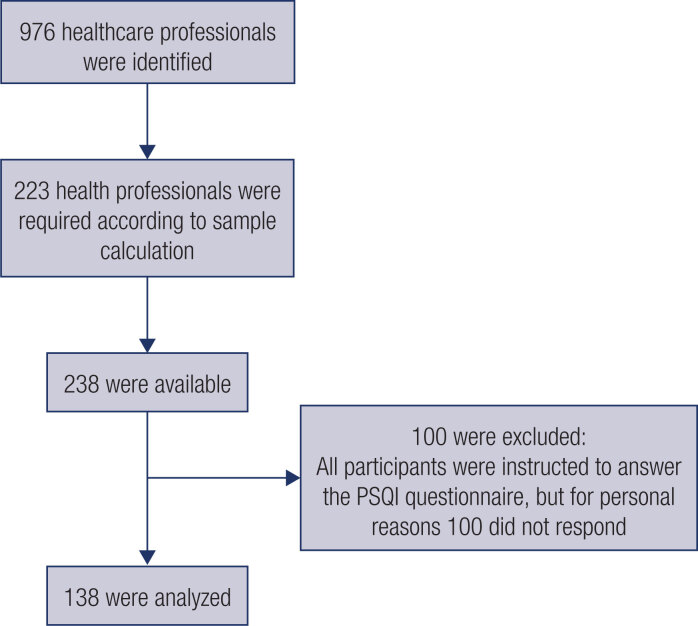
Flowchart of origin of data used in this study.

As a baseline, we surveyed 976 healthcare professionals in Viçosa, Brazil. The calculated sample size was 223 subjects, with a 95% confidence interval (CI), 5% sampling error, and an expected metabolic syndrome prevalence of 25%. However, our sample size is contingent on the subset of participants who had completed the Pittsburgh sleep quality index (PSQI) questionnaire (N = 138; Figure 1). Supplementary Table 1 shows the characteristics of participants who had and had not completed the PSQI questionnaire. Subjects who had not completed the questionnaire showed higher TyG index and very-low-density lipoprotein cholesterol (VLDL-c) values than those who completed the PSQI questionnaire.

Assuming a prevalence of TyG index > 8.08 or > 4.38 in the exposed (poor sleep quality) and non-exposed (good sleep quality) groups, respectively, the analysis had 89.49% power to detect a difference of this magnitude or larger, determined using the OpenEpi online software (28).

### Collected data

#### Sleep Quality (Exposure)

Sleep quality was assessed by the adapted and validated Brazilian version of the PSQI (29). The information refers to the last month and contains nineteen items that cover seven components: subjective sleep quality (contentment at daily sleep), sleep latency (extended sleep onset time), sleep duration, habitual sleep efficiency (proportion of hours slept relative to total hours in bed), sleep disorders (disruption of sleep), use of sleeping medication, and daytime dysfunction (difficulty staying awake during social activities) (30). We assessed the global PSQI score from 0 to 21 points (30). Finally, volunteers were categorized into two groups: good sleep quality (global PSQI score ≤ 5) or poor sleep quality (global PSQI score ≥ 6).

#### Dietary intake assessment and lifestyle

Dietary intake was assessed by a semi-quantitative food frequency questionnaire, validated for a Spanish population, and adapted for Brazilian citizens, with 136 food items (31). Nutrient intake was estimated using ad hoc computer software specifically developed for this aim. In addition, updated information from Brazilian food composition tables was considered. A trained professional was responsible for administering the questionnaire to minimize potential bias. To evaluate physical activity, we used the international physical activity questionnaire (IPAQ), which is validated for the Brazilian population (32). Smoking habit was determined by asking the participants whether they were smokers, former smokers, or nonsmokers.

#### Anthropometric, body composition, and blood pressure

Weight and height were measured to calculate body mass index (BMI) by dividing the weight (kg) by height (m) squared. Overweight was classified as BMI ≥ 25 kg/m². Waist circumference (WC) was measured at the midpoint between the last rib and the iliac crest using a flexible and inelastic tape measure. Hip circumference (HC) was measured in the greater protuberance in the gluteal region. The waist-to-height ratio was calculated as the ratio of WC (cm) and height (cm). Body composition was evaluated with a BMI 310 Bioimpedance Analyzer (Biodynamic Research Corporation; San Antonio, TX, USA) according to standardized measurement conditions (27). The systolic and diastolic blood pressures were measured using an Omron HEM-742INT digital sphygmomanometer (Hoffman Estates, IL, USA) according to the protocol recommended by the European Society of Hypertension and the European Society of Cardiology (33). Research team members were suitably trained to obtain these measurements.

#### Metabolic markers (Outcomes)

Venous blood samples were drawn following a 12-hour fast, centrifuged at 3500 rpm at 4 °C for 10 min (Megafuge 11R; Thermo Scientific, Waltham, MA, USA), and stored at -80 °C. A trained health professional was responsible for the blood collection. Triglyceride, total cholesterol, high-density lipoprotein cholesterol (HDL-c), and uric acid levels were determined by the enzymatic colorimetric method. Fasting glycemia was determined by the glucose oxidase method. Friedewald’s equation was used to calculate values of low-density lipoprotein cholesterol (LDL-c) and VLDL-c (34). The TyG index was calculated as Ln [triglyceride (mg/dL) × glycemia (mg/dL)/2] and as Ln [triglycerides (mg/dL) × glycemia (mg/dL)]/2. We performed the calculation using both formulas because of discrepancies in TyG index cutoff values observed in the literature (35). The final division in the TyG formula is applied outside of the square brackets by some studies and not by other studies (35). Complement component 3 (C3) was obtained by the immunoturbidimetry method, and C-Reactive Protein (CRP) levels were determined by enzyme-linked immunosorbent assays (ELISA; Multiskan FC; Thermo Scientific) with the ultra-sensitive DSL-C-reactive protein kit. Plasma levels of different cytokines, tumor necrosis factor (TNF), interleukins (IL-1b, IL-6, IL-10), and adiponectin were determined by ELISA multiplex using a commercial kit (Biosource [Camarillo, CA, USA] or Sellex [São Paulo, Brazil]).

#### Statistical analysis

Statistical analyzes were performed using MedCalc (v.9.3; Ostend, Belgium) and the R statistical software (v.4.1.0). Subjects were classified into two groups according to the global PSQI score: good sleep quality (≤5) and poor sleep quality (≥6). Subjects with missing PSQI data were excluded from the analyses. A cutoff value for the TyG index was estimated, taking the presence of IR (HOMA-IR>2.71) as a reference (36). Then, the area under the curve (AUC) for receiving operating characteristic (ROC) curves was calculated to obtain sensitivity and specificity estimates. Variable normality was assessed by the Shapiro-Wilk test. The Student’s *t-test* or Pearson Chi-square test was used to compare the subject characteristics according to the TyG index cutoff values. The data are presented as mean with standard deviation (SD) and frequencies. The prevalence of subjects with TyG index values above the cutoff was greater than 10% in our data. For this reason, Poisson regression with robust variance was used to assess the association between the TyG index (categorical and dependent variable) and the sleep quality (categorical and independent variable). The variables associated with the TyG index by the hypothesis test were used to adjust the regression analysis. The variable VLDL-c was calculated using the triglyceride values. Therefore, we do not include it as an adjustment variable. The data are presented as prevalence ratio (95% CI). A 5% significance level was used for all tests performed.

## RESULTS

**Figure 2:**
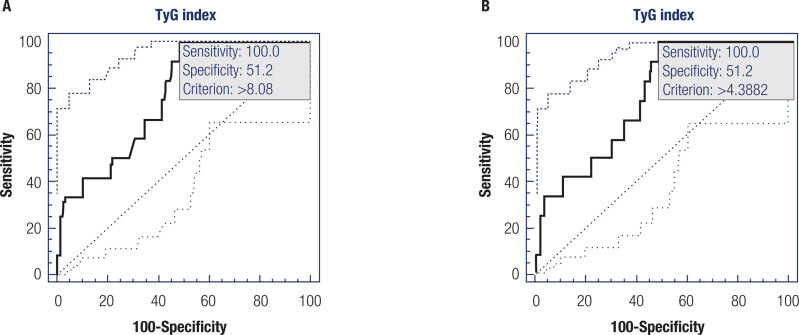
Cut-off for TyG index based on the presence of insulin resistance based on HOMA-IR. (**A**) TyG index calculated through the formula: Ln [triglycerides (mg/dL) * glycemia (mg/dL)/2]; (**B**) TyG index calculated through the formula: Ln [triglycerides (mg/dL) * glycemia (mg/dL)]/2. Area under the curve (AUC) = 0.766, Standard error = 0.0825, 95 % Confidence interval 95% = 0.685-0.834, z-statistic = 3.224, significance level p (Area=0.5) = 0.0013.

Of the 138 subjects included in the study, 103 (75.7 %) were female with a mean age of 29.17 (SD = 7.23) years, 39.9 % presented poor sleep quality evaluated through PSQI, and 8.9% presented IR evaluated by HOMA-IR. We estimated cutoffs for the TyG index, taking IR presence as a reference. Therefore, subjects were classified as having a TyG index of ≤8.08 or >8.08, or ≤4.38 or >4.38 (Figure 2). 100% sensitivity and 51.2% specificity were found for the optimal TyG index cutoff (Supplementary Tables 3 and 4).

**Figure 3:**
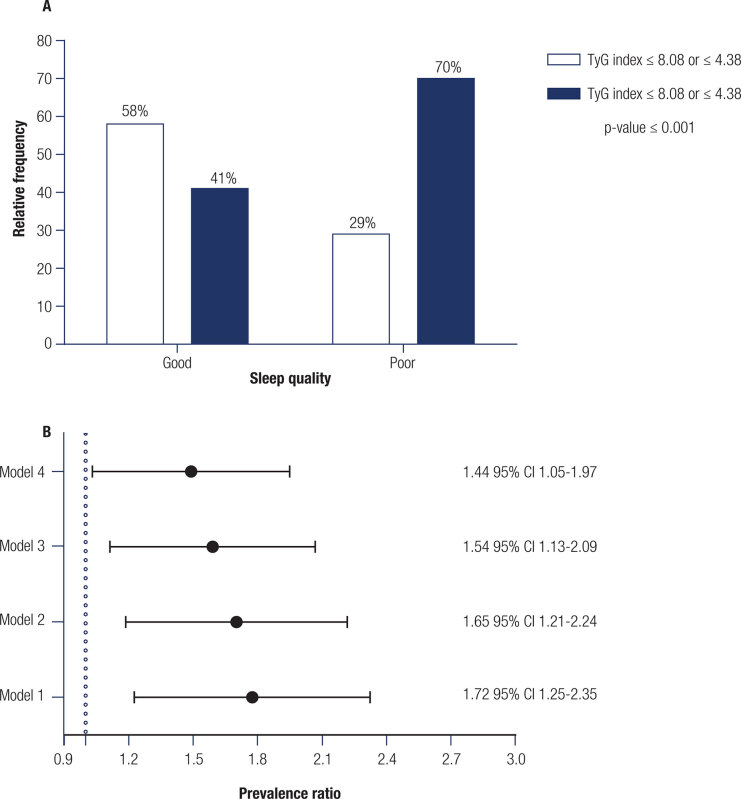
Association between the sleep quality measured by Pittsburgh Sleep Quality Index (PSQI) and cut-off values of TyG index. Good sleep quality: PSQI score ≤ 5 points; and poor sleep quality: PSQI score ≥ 6 points. **A.** Prevalence of subjects with good or poor sleep quality according to cut-off values of TyG index. Data are presented as relative frequency. P-value obtained through Pearson Chi-square test. **B.** Prevalence ratio of subjects classified with TyG index > 8.08 or > 4.38 and poor sleep quality. Data are prevalence ratio and 95% CI obtained through Poisson regression with robust variance. Reference category: Subjects classified as having good sleep quality. **Model 1:** Crude; **Model 2:** Adjusted by sex, total cholesterol and LDL/HDL ratio; **Model 3:** Adjusted by variables in model 2 + insulin; **Model 4:** Adjusted by variables in model 3 + C3 complement, and C-reactive protein + Adiponectin.

Higher levels of total cholesterol, VLDL-c, LDL-c, LDL/HDL ratio, insulin, complement C3, CRP, and lower adiponectin levels were found in subjects with TyG index >8.08 or >4.38 (Table 1). Characteristics of the participants according to sleep quality are presented in Supplementary Table 2. Briefly, subjects with poor sleep quality were mostly male and presented higher values of muscle mass, VLDL-c, insulin, and TyG index but lower percentages of body fat and carbohydrate intake than those with good sleep quality. However, while most subjects (70%) with high TyG index values (>8.08 or >4.38) were likely to have poor sleep quality, 41% had good sleep quality (Figure 3A). We found that subjects with poor sleep quality had a 1.44-fold higher prevalence of IR (TyG index > 8.08 or > 4.38) compared to those with good sleep quality, regardless of sex and total cholesterol, LDL/HDL ratio, insulin, complement C3, CRP, and adiponectin levels (Figure 3B).

**Table 1:** General characteristics of the subjects according to TyG index cut-off values

		TyG index cut-off values		p-value
		≤8.08 or ≤4.38	>8.08 or >4.38	
Age, years		28.11 (5.63)	29.79 (7.94)	0.158
Sex				
	Women	50 (49)	52 (51)	0.423
	Men	13 (39.4)	20 (60.6)	
Physical activity				
	Active	55 (46.6)	63 (53.4)	1.000
	Non active	8 (47.1)	9 (52.9)	
Smoking habit				
	Non-smokers	56 (44.8)	69 (55.2)	0.412
	Smoker + former smokers	4 (66.7)	2 (33.3)	
Waist circumference, cm		77.67 (8.33)	79.91 (10.16)	0.168
Hip circumference, cm		98.47 (7.43)	99.08 (5.09)	0.574
Waist-to-hip ratio		0.78 (0.06)	0.8 (0.07)	0.237
Body mass index, kg/m²		22.18 (3.11)	22.8 (3.31)	0.270
Body fat, %		21.93 (6.52)	22.96 (5.84)	0.336
Body fat, kg		13.57 (5.24)	14.96 (4.89)	0.114
Muscle mass, kg		48.13 (9.74)	50.42 (10.8)	0.201
PAS, mmHg		107.42 (11.5)	110.4 (13.63)	0.175
PAD, mmHg		65.46 (6.42)	68 (8.59)	0.056
Cardiac frequency,		71.66 (15.1)	75 (10.21)	0.130
Total cholesterol, mg/dL		171.57 (31.69)	201.14 (33.48)	<0.001
HDL-c, mg/dL		58.25 (12.91)	59.79 (17.31)	0.557
VLDL-c, mg/dL		11.64 (2.51)	22.93 (7.08)	<0.001
LDL-c, mg/dL		101.67 (27.98)	117.89 (27.57)	0.001
LDL/HDL		1.83 (0.65)	2.13 (0.87)	0.025
Insulin, μUI/mL		5.54 (2.57)	8.73 (5.82)	<0.001
HOMA-IR		1.14 (0.53)	2.10 (2.60)	0.003
Uric acid, g/mL		4.1 (1.19)	4.11 (1.25)	0.955
Complement C3, mg/dL		95.74 (14.74)	111.29 (18.28)	<0.001
CRP, mg/L		1.83 (2.49)	3.74 (5.8)	0.015
Adiponectin, mcg/mL		15.52 (8.13)	12.82 (5.6)	0.029
Interleukin-10, pg/mL		1.85 (0.83)	1.88 (0.92)	0.881
Interleukin-1b, pg/mL		1.16 (0.4)	1.2 (0.36)	0.545
Interleukin-6, pg/mL		1.45 (1.28)	1.27 (0.53)	0.301
TNF, pg/mL		6.55 (2.74)	6.7 (2.36)	0.741
Calories, kcal		2,586.92 (799.93)	2,594.55 (694.43)	0.953
Carbohydrates, %		52.65 (12.48)	50.52 (11.6)	0.305
Proteins, %		18.18 (6.42)	17.32 (4.11)	0.354
Fibers, mg/dL		53.33 (45.2)	42.45 (23.69)	0.090
Sodium, mg		2,501.81 (1,657.57)	2,775.45 (1,849.62)	0.370
Alcohol consumption, %		4.16 (13.88)	9.85 (25.09)	0.103
Lipids, %		31.81 (10.38)	32.62 (9.89)	0.641
Cholesterol, mg/dL		386.32 (339.94)	373.23 (213.19)	0.786
SFA, %		11.84 (6.14)	12.5 (5.32)	0.509
MUFA, %		10.88 (5.29)	10.38 (3.59)	0.524
PUFA, %		2.37 (1.62)	2.5 (1.56)	0.613

Data are presented as mean (SD) for quantitative variables and as frequency absolute (frequency relative) for categorical variables. P-values were obtained through Student-*t*-test or Pearson Chi-square test.

CRP: C-reactive protein; DBP: diastolic blood pressure; HDL-c: high-density lipoprotein cholesterol; HOMA-IR: homeostatic model assessment for IR; LDL-c: low-density lipoprotein cholesterol; MUFA: monounsaturated fatty acid; PUFA: polyunsaturated fatty acid; SBP: systolic blood pressure; SFA: saturated fatty acid; TyG index: triglyceride-glucose index; TNF: tumor necrosis factor; VLDL-c: very low-density lipoprotein cholesterol.

**Figure 4:**
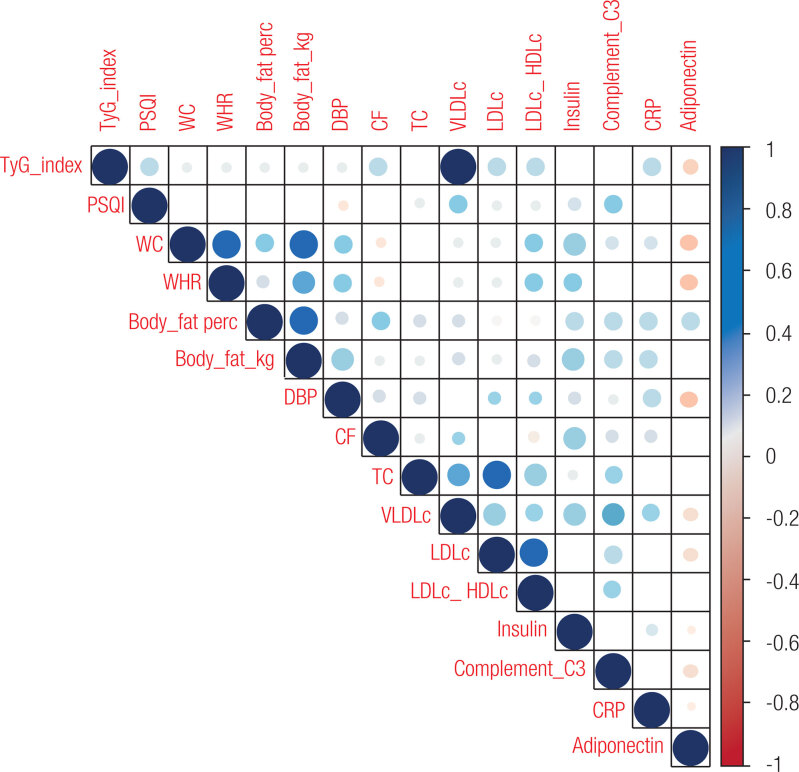
Correlation matrix of variables related to the TyG index. All variables have at least a statistically significant correlation with the TyG index in Pearson’s chi-square test. R coefficient interpretation: positive values = direct correlation; negative values = inverse correlation; 0 = absence of correlation; <0.30 = weak correlation; ≤0.30 and ≤70 = moderate correlation; >70 = strong correlation. TyG_index: triglyceride-glucose index; PSQI: Pittsburgh Sleep Quality Index; WC: waist circumference; WHR: waist-hip ratio; Body_fat_perc: percentage of body fat; Body_fat_kg: kilograms of body fat; DBP: diastolic blood pressure; CF: cardiac frequency; TC, total cholesterol; VLDL-c: very-low-density lipoprotein cholesterol; LDLc: low-density lipoprotein cholesterol; LDLc_HDLc: LDLc HDLc ratio; Complement_C3: complement component 3; CRP: C-reactive protein.

Finally, we constructed a correlation matrix to explore the correlations of variables related to TyG index. TyG index was positively correlated with the PSQI score, waist circumference, waist-to-height ratio, body fat, diastolic blood pressure, cardiac frequency, total cholesterol, insulin, complement C3, and CRP. Conversely, higher TyG values were negatively associated with adiponectin levels (Figure 4).

## DISCUSSION

To the best of our knowledge, this is the first study to evaluate the association between the TyG index and sleep quality in ostensibly healthy adults. The two previous cross-sectional studies have associated TyG with OSA (25,26) but did not consider sleep quality. We found that subjects with poor sleep quality had a 1.44-fold higher risk of having a TyG index above the cutoff than those with good sleep quality, regardless of sex and total cholesterol, LDL/HDL ratio, insulin, complement C3, CRP, and adiponectin levels. A number of established mechanisms have addressed the link between metabolic disorder and IR, defined as a state that stimulates impairments in glucose uptake, particularly glycogen synthesis (2). This metabolic disorder causes hyperglycemia and leads to oxidative stress and inflammatory responses (2). IR also leads to dyslipidemia because adipocytes increase their release of free fatty acids, which are absorbed by the liver to form triglyceride-rich and VLDL-c particles in large circulating amounts (2,36).

Evidence has shown that sleep fragmentation can change glucose metabolism by reducing insulin sensitivity (19). Endocrine mechanisms underlie the influence of sleep on IR through inflammatory pathways and persistent activation of the sympathetic and HPA axis (37). Poor sleep quality has been associated with high inflammatory marker (38) and cortisol (39) levels. Sympathetic and HPA axis activation has been reported to increase catecholamine and cortisol secretion (37). Combined with a proinflammatory state, these factors could contribute to IR development. Additionally, poor sleep quality seems to have epigenetic effects and share genetic architecture with metabolic syndrome (40). A study found a genetic correlation between insomnia symptoms and HOMA-IR, suggesting the involvement of genetic variants (41).

Current data have indicated a close relationship between HOMA-IR and TyG index (22,42). In Brazil, a validated study concluded that the TyG index had a better performance than the HOMA-IR index for measuring IR in clinical practice (22). Furthermore, a population-based cross-sectional study found a correlation between TyG and other markers of IR, such as HOMA-IR and the hyperinsulinemic-euglycemic clamp (HIEC), in healthy subjects (42). A systematic review has found that the highest achieved sensitivity was 96% using HIEC (43). The highest specificity was 99% using HOMA-IR, with a cutoff value of 4.68 (43), close to the value of 4.38 in this study.

We estimated TyG index cutoffs to detect IR based on two different methods for calculating the TyG index. While the formulas are identical, some studies have applied the division outside of the square brackets, and others have not. Therefore, the TyG index cutoff values reported in the literature range from ~4 and ~8 (35). Kang and cols. (25) recently reported a cut-off for the TyG index of 8.83 (sensitivity: 61.6%; specificity: 69.0%; AUC: 0.688;*P* = 0.001) among subjects with suspected OSA. To estimate our cutoff values, we used HOMA-IR > 2.71 as a reference according to the Brazilian guidelines for diabetes (36). In this study, the TyG index > 8.08 or > 4.38 was the optimal value to identify IR in our samples, with 100% sensitivity and 51.2% specificity. These values suggest that subjects with a TyG index > 8.08 or > 4.38 have IR, with 0% false-positive cases. However, the TyG index is not a good measure to detect subjects without IR, with a high false-negative rate.

Previous studies have reported a positive association between the TyG index, IR, and related conditions such as T2D (24) and cardiovascular events (44). Subjects in our study with IR determined by a TyG index > 8.08 or > 4.38 had worse metabolic profiles, with higher total cholesterol, VLDL-c, LDL-c, LDL/HDL ratio, insulin, complement C3, and CRP values and lower adiponectin levels than those with a TyG index lower ≤ 8.08 or ≤ 4.38. Moreover, we found simultaneous and positive correlations between the TyG index and cardiometabolic risk variables such as waist circumference, waist-to-height ratio, body fat, diastolic blood pressure, cardiac frequency, total cholesterol, and fractions (including VLDL-c, LDL-c and LDL/HDL ratio), insulin, complement C3, and CRP. These observations are consistent with previous studies that found a worse metabolic profile in subjects classified in the highest quartiles of the TyG index (24,44).

Poor sleep quality and IR could contribute to chronic inflammation, but it remains challenging to manipulate factors such as diet and sleep that may affect inflammation experimentally (45). Complement C3 and CRP are prominent biomarkers for IR (46,47). As mentioned above, subjects with a TyG index > 8.08 or > 4.38 had higher values for these inflammation markers. Uemura and cols. have reported that higher serum CRP was associated with IR in a dose-dependent manner (47). In another study, complement C3 was strongly associated with IR, independent of the other components of metabolic syndrome (46). Moreover, our results have shown a simultaneous correlation between the TyG index and adiponectin, a crucial modulator of insulin sensitivity and chronic inflammation (48).

This study used a cutoff value for the TyG index > 8.08 or > 4.38 as a surrogate marker to estimate IR. It was associated with poor sleep quality among apparently healthy adults, and its predictive significance also correlates with other important independent risk factors. Although the prevalence in women and men was not statistically different between the sleep quality and TyG index categories, selection bias potentially limits our study because of the high frequency of females in our data. In addition, since it is a cross-sectional study, it cannot establish a causal relationship. While the HOMA-IR test is not the gold standard for diagnosing IR, the euglycemic-hyperinsulinemic clamp is the gold standard, but it is impractical for use in large cohort studies. The PSQI questionnaire was available for all subjects in the study. However, 100 subjects did not respond to the questionnaire, creating additional selection biases in our study.

This study’s greatest benefit is its use of ostensibly healthy adults before the onset of chronic diseases to highlight the potential involvement of poor sleep quality in IR, even before it manifests clinically. Our findings reinforce the need for further research into using the TyG index as a surrogate marker of IR and its relationship with sleep. In addition, understanding modifiable risk factors for IR in adults may offer more effective primary prevention efforts in an at-risk population and expansion of interventions to improve sleep quality.

## SUPPLEMENTARY MATERIALS

**Supplementary Table 1:** General characteristics of the subjects according to completion of the Pittsburgh Sleep Quality Index (PSQI) score

		Completed PSQI questionnaire		p-value
		Yes (n = 138)	No (n = 100)	
Age, years*		29.17 (7.24)	28.92 (8.07)	0.811
Sex				
	Women	103 (75.7)	65 (72.2)	0.641
	Men	33 (24.3)	25 (27.8)	
Physical activity				
	Active	119 (87.5)	73 (83.9)	0.552
	Non active	17 (12.5)	14 (16.1)	
Smoking habit				
	Non-smokers	128 (97)	74 (96.1)	0.710
	Smoker + former smokers	4 (3)	3 (3.9)	
Waist circumference, cm		78.94 (9.4)	79.34 (10.68)	0.771
Hip circumference, cm		98.81 (6.25)	98.57 (6.92)	0.785
Waist-to-hip ratio		0.80 (0.07)	0.80 (0.08)	0.651
Body mass index, kg/m²		22.52 (3.21)	22.95 (3.75)	0.352
Body fat, %		22.54 (6.18)	22.81 (6.99)	0.758
Body fat, kg		14.35 (5.09)	14.71 (6.55)	0.647
Muscle mass, kg		49.31 (10.32)	49.64 (11.39)	0.826
PAS, mmHg		109.02 (12.68)	108.90 (11.9)	0.944
PAD, mmHg		66.84 (7.71)	67.73 (7.74)	0.399
Cardiac frequency		73.38 (12.76)	73.18 (10.39)	0.899
Total cholesterol, mg/dL		187.34 (35.75)	178.84 (36.99)	0.087
HDL-c, mg/dL		59.07 (15.38)	59.69 (15.15)	0.770
VLDL-c, mg/dL		17.63 (7.83)	20.16 (9.23)	0.028
LDL-c, mg/dL		110.27 (28.83)	99.04 (32.71)	0.007
LDL/HDL		1.99 (0.79)	1.77 (0.72)	0.036
Insulin, μUI/mL		7.25 (4.86)	7.34 (3.77)	0.876
HOMA-IR		1.65 (1.99)	1.61 (0.91)	0.848
Triglycerides, mg/dL		91.2 (52.9)	100.82 (46.17)	0.163
Glucose, mg/dL		87.17 (13.99)	87.73 (7.39)	0.729
TyG index 1		8.17 (0.47)	8.30 (0.45)	0.040
TyG index 2		4.43 (0.23)	4.49 (0.22)	0.040
Uric acid, g/mL		4.11 (1.22)	4.00 (1.09)	0.499
Complement C3, mg/dL		104.22 (18.42)	107.38 (18.15)	0.228
CRP, mg/L		2.85 (4.64)	3.52 (7.84)	0.430
Adiponectin, mcg/mL		14.09 (7.01)	16.31 (9.16)	0.042
Interleukin-10, pg/mL		1.87 (0.88)	1.83 (0.89)	0.732
Interleukin-1b, pg/mL		1.19 (0.38)	1.09 (0.16)	0.016
Interleukin-6, pg/mL		1.36 (0.96)	1.40 (0.83)	0.717
TNF, pg/mL		6.63 (2.54)	6.99 (3.5)	0.377
Calories, kcal		2596.67 (738.63)	2454.94 (819.77)	0.174
Carbohydrates, %		51.69 (11.98)	51.21 (13.85)	0.782
Proteins, %		17.72 (5.28)	18.12 (6.48)	0.605
Fibers, mg/dL		47.77 (35.4)	41.33 (24.46)	0.130
Sodium, mg		2,677.90 (1819.44)	2,348.77 (2001.37)	0.198
Alcohol consumption, %		7.15 (20.5)	4.77 (11.56)	0.313
Lipids, %		32.07 (10.13)	32.97 (14.25)	0.577
Cholesterol, mg/dL		378.42 (275.66)	432.57 (612.34)	0.428
SFA, %		12.08 (5.71)	13.07 (11.32)	0.382
MUFA, %		10.53 (4.47)	10.86 (4.69)	0.588
PUFA, %		2.46 (1.6)	2.51 (1.7)	0.822

Data are presented as mean (SD) for quantitative variables and as frequency absolute (frequency relative) for categorical variables. P-values were obtained through Student-*t*-test or Pearson Chi-square test. TyG index 1 was calculated through the formula Ln [triglyceride (mg/dL) x glycemia (mg/dL)/2]. TyG index 2 was calculated through the formula Ln [triglycerides (mg/dL) x glycemia (mg/dL)]/2. CRP: C-reactive protein; DBP: diastolic blood pressure; HDL-c: high-density lipoprotein cholesterol; HOMA-IR: homeostatic model assessment for IR; LDL-c: low-density lipoprotein cholesterol; MUFA: monounsaturated fatty acid; PUFA: polyunsaturated fatty acid; SBP: systolic blood pressure; SFA: saturated fatty acid; TyG index: Triglyceride-glucose index; TNF: tumor necrosis factor; VLDL-c: very low-density lipoprotein cholesterol.

**Supplementary Table 2:** General characteristics of the subjects based on the Pittsburgh Sleep Quality Index (PSQI) score

		Sleep quality		p-value
		Good	Poor	
Age, years		29.40 (7.08)	28.84 (7.51)	0.664
Sex				
	Women	68 (66)	35 (34)	0.008
	Men	13 (39.4)	20 (60.6)	
Physical activity				
	Active	68 (57.1)	51 (42.9)	0.187
	Non active	13 (76.5)	4 (23.5)	
Smoking habit				
	Non-smokers	75 (60)	50 (40)	0.443
	Smoker + former smokers	3 (42.9)	4 (57.1)	
Waist circumference, cm		78.83 (9.21)	79.11 (9.75)	0.862
Hip circumference, cm		98.65 (7.13)	99.05 (4.73)	0.691
Waist-to-hip ratio		0.80 (0.07)	0.80 (0.08)	0.905
Body mass index, kg/m²		22.41 (3.40)	22.67 (2.94)	0.642
Body fat, %		23.41 (5.82)	21.26 (6.51)	0.047
Body fat, kg		14.68 (5.14)	13.86 (5.01)	0.357
Muscle mass, kg		47.60 (9.04)	51.83 (11.59)	0.025
PAS, mmHg		108.31 (12.11)	110.06 (13.53)	0.431
PAD, mmHg		66.93 (8.23)	66.71 (6.95)	0.872
Cardiac frequency,		74.01 (14.04)	72.46 (10.66)	0.490
Total cholesterol, mg/dL		185.45 (36.75)	190.09 (34.39)	0.461
HDL-c, mg/dL		59.35 (14.44)	58.67 (16.79)	0.803
VLDLc, mg/dL		15.97 (6.90)	20.00 (8.53)	0.003
LDL-c, mg/dL		109.47 (29.09)	111.42 (28.68)	0.702
LDL/HDL		1.92 (0.64)	2.09 (0.96)	0.262
Insulin, μUI/mL		6.56 (3.64)	8.25 (6.12)	0.046
HOMA-IR		1.41 (0.85)	2.00 (2.92)	0.089
TyG index 1		8.09 (0.48)	8.29 (0.43)	0.014
TyG index 2		4.39 (0.24)	4.49 (0.21)	0.014
Uric acid, g/mL		3.95 (1.18)	4.37 (1.25)	0.054
Complement C3, mg/dL		102.01 (17.51)	107.55 (19.43)	0.103
CRP, mg/L		3.01 (5.20)	2.59 (3.66)	0.610
Adiponectin, mcg/mL		14.59 (6.86)	13.35 (7.21)	0.315
Interleukin-10, pg/mL		1.90 (0.95)	1.83 (0.78)	0.655
Interleukin-1b, pg/mL		1.19 (0.42)	1.18 (0.32)	0.870
Interleukin-6, pg/mL		1.44 (1.16)	1.25 (0.55)	0.261
TNF, pg/mL		6.72 (2.69)	6.51 (2.31)	0.636
Calories, kcal		2,591.70 (764.40)	2,604.17 (704.83)	0.923
Carbohydrates, %		53.46 (11.84)	49.01 (11.79)	0.032
Proteins, %		17.45 (4.96)	18.12 (5.75)	0.467
Fibers, mg/dL		50.77 (39.94)	43.25 (26.88)	0.223
Sodium, mg		2,579.39 (1,849.05)	2,826.56 (1,780.26)	0.437
Alcohol consumption, %		4.53 (15.26)	11.05 (26.14)	0.099
Lipids, %		31.40 (10.01)	33.09 (10.32)	0.338
Cholesterol, mg/dL		363.34 (294.17)	401.17 (245.96)	0.432
SFA, %		11.84 (5.98)	12.44 (5.31)	0.549
MUFA, %		10.53 (4.79)	10.53 (3.97)	0.998
PUFA, %		2.34 (1.58)	2.64 (1.63)	0.281

Data are presented as mean (SD) for quantitative variables and as frequency absolute (frequency relative) for categorical variables. P-values were obtained through Student-t-test or Pearson Chi-square test. TyG index 1 was calculated through the formula Ln [triglyceride (mg/dL) x glycemia (mg/dL)/2]. TyG index 2 was calculated through the formula Ln [triglycerides (mg/dl) x glycemia (mg/dL)]/2. CRP: C-reactive protein; DBP: diastolic blood pressure; HDL-c: high-density lipoprotein cholesterol; HOMA-IR: homeostatic model assessment for IR; LDL-c: low-density lipoprotein cholesterol; MUFA: monounsaturated fatty acid; PUFA, polyunsaturated fatty acid; SBP: systolic blood pressure; SFA: saturated fatty acid; TyG index: triglyceride-glucose index; TNF: tumor necrosis factor; VLDL-c: very low-density lipoprotein cholesterol.

**Supplementary Table 3:** Criterion values and coordinates of the ROC curve

Criterion	Sensitivity	95% CI	Specificity	95% CI	+LR	-LR	+PV	-PV
≥7.33	100.00	73.4-100.0	0.00	0.0-3.0	1.00		8.9	
>7.33	100.00	73.4-100.0	0.81	0.1-4.5	1.01	0.00	9.0	100.0
>7.35	100.00	73.4-100.0	3.25	0.9-8.1	1.03	0.00	9.2	100.0
>7.41	100.00	73.4-100.0	4.07	1.3-9.2	1.04	0.00	9.2	100.0
>7.44	100.00	73.4-100.0	4.88	1.8-10.3	1.05	0.00	9.3	100.0
>7.45	100.00	73.4-100.0	5.69	2.3-11.4	1.06	0.00	9.4	100.0
>7.48	100.00	73.4-100.0	6.50	2.9-12.4	1.07	0.00	9.4	100.0
>7.5	100.00	73.4-100.0	7.32	3.4-13.4	1.08	0.00	9.5	100.0
>7.53	100.00	73.4-100.0	8.13	4.0-14.4	1.09	0.00	9.6	100.0
>7.56	100.00	73.4-100.0	8.94	4.6-15.4	1.10	0.00	9.7	100.0
>7.58	100.00	73.4-100.0	9.76	5.1-16.4	1.11	0.00	9.8	100.0
>7.6	100.00	73.4-100.0	10.57	5.8-17.4	1.12	0.00	9.8	100.0
>7.61	100.00	73.4-100.0	11.38	6.4-18.4	1.13	0.00	9.9	100.0
>7.62	100.00	73.4-100.0	12.20	7.0-19.3	1.14	0.00	10.0	100.0
>7.63	100.00	73.4-100.0	13.82	8.3-21.2	1.16	0.00	10.2	100.0
>7.64	100.00	73.4-100.0	14.63	8.9-22.1	1.17	0.00	10.3	100.0
>7.66	100.00	73.4-100.0	15.45	9.6-23.1	1.18	0.00	10.3	100.0
>7.67	100.00	73.4-100.0	16.26	10.2-24.0	1.19	0.00	10.4	100.0
>7.68	100.00	73.4-100.0	19.51	12.9-27.6	1.24	0.00	10.8	100.0
>7.7	100.00	73.4-100.0	21.14	14.3-29.4	1.27	0.00	11.0	100.0
>7.74	100.00	73.4-100.0	21.95	15.0-30.3	1.28	0.00	11.1	100.0
>7.78	100.00	73.4-100.0	24.39	17.1-33.0	1.32	0.00	11.4	100.0
>7.79	100.00	73.4-100.0	26.83	19.2-35.6	1.37	0.00	11.8	100.0
>7.8	100.00	73.4-100.0	28.46	20.7-37.3	1.40	0.00	12.0	100.0
>7.81	100.00	73.4-100.0	29.27	21.4-38.2	1.41	0.00	12.1	100.0
>7.82	100.00	73.4-100.0	30.89	22.9-39.9	1.45	0.00	12.4	100.0
>7.84	100.00	73.4-100.0	31.71	23.6-40.7	1.46	0.00	12.5	100.0
>7.87	100.00	73.4-100.0	32.52	24.4-41.6	1.48	0.00	12.6	100.0
>7.88	100.00	73.4-100.0	34.15	25.8-43.2	1.52	0.00	12.9	100.0
>7.89	100.00	73.4-100.0	34.96	26.6-44.1	1.54	0.00	13.0	100.0
>7.9	100.00	73.4-100.0	35.77	27.3-44.9	1.56	0.00	13.2	100.0
>7.91	100.00	73.4-100.0	36.59	28.1-45.7	1.58	0.00	13.3	100.0
>7.92	100.00	73.4-100.0	37.40	28.8-46.6	1.60	0.00	13.5	100.0
>7.93	100.00	73.4-100.0	39.02	30.4-48.2	1.64	0.00	13.8	100.0
>7.94	100.00	73.4-100.0	39.84	31.1-49.1	1.66	0.00	14.0	100.0
>7.96	100.00	73.4-100.0	40.65	31.9-49.9	1.68	0.00	14.1	100.0
>7.98	100.00	73.4-100.0	41.46	32.7-50.7	1.71	0.00	14.3	100.0
>7.99	100.00	73.4-100.0	43.90	35.0-53.1	1.78	0.00	14.8	100.0
>8	100.00	73.4-100.0	44.72	35.7-53.9	1.81	0.00	15.0	100.0
>8.02	100.00	73.4-100.0	47.15	38.1-56.4	1.89	0.00	15.6	100.0
>8.06	100.00	73.4-100.0	48.78	39.7-58.0	1.95	0.00	16.0	100.0
>8.07	100.00	73.4-100.0	50.41	41.3-59.5	2.02	0.00	16.4	100.0
>8.08 *	100.00	73.4-100.0	51.22	42.0-60.3	2.05	0.00	16.7	100.0
>8.09	91.67	61.5-98.6	51.22	42.0-60.3	1.88	0.16	15.5	98.4
>8.1	91.67	61.5-98.6	53.66	44.4-62.7	1.98	0.16	16.2	98.5
>8.12	91.67	61.5-98.6	54.47	45.2-63.5	2.01	0.15	16.4	98.5
>8.14	83.33	51.6-97.4	55.28	46.1-64.3	1.86	0.30	15.4	97.1
>8.15	83.33	51.6-97.4	56.10	46.9-65.0	1.90	0.30	15.6	97.2
>8.17	83.33	51.6-97.4	56.91	47.7-65.8	1.93	0.29	15.9	97.2
>8.18	75.00	42.8-94.2	57.72	48.5-66.6	1.77	0.43	14.8	95.9
>8.19	75.00	42.8-94.2	58.54	49.3-67.3	1.81	0.43	15.0	96.0
>8.2	66.67	34.9-89.9	58.54	49.3-67.3	1.61	0.57	13.6	94.7
>8.21	66.67	34.9-89.9	59.35	50.1-68.1	1.64	0.56	13.8	94.8
>8.22	66.67	34.9-89.9	60.16	50.9-68.9	1.67	0.55	14.0	94.9
>8.25	66.67	34.9-89.9	60.98	51.8-69.6	1.71	0.55	14.3	94.9
>8.26	66.67	34.9-89.9	61.79	52.6-70.4	1.74	0.54	14.5	95.0
>8.27	66.67	34.9-89.9	62.60	53.4-71.2	1.78	0.53	14.8	95.1
>8.28	66.67	34.9-89.9	63.41	54.3-71.9	1.82	0.53	15.1	95.1
>8.29	66.67	34.9-89.9	65.04	55.9-73.4	1.91	0.51	15.7	95.2
>8.31	58.33	27.8-84.7	65.04	55.9-73.4	1.67	0.64	14.0	94.1
>8.32	58.33	27.8-84.7	65.85	56.8-74.2	1.71	0.63	14.3	94.2
>8.34	58.33	27.8-84.7	67.48	58.4-75.6	1.79	0.62	14.9	94.3
>8.36	58.33	27.8-84.7	68.29	59.3-76.4	1.84	0.61	15.2	94.4
>8.38	58.33	27.8-84.7	69.11	60.1-77.1	1.89	0.60	15.6	94.4
>8.4	50.00	21.2-78.8	71.54	62.7-79.3	1.76	0.70	14.6	93.6
>8.41	50.00	21.2-78.8	72.36	63.6-80.0	1.81	0.69	15.0	93.7
>8.43	50.00	21.2-78.8	73.17	64.4-80.8	1.86	0.68	15.4	93.7
>8.45	50.00	21.2-78.8	73.98	65.3-81.5	1.92	0.68	15.8	93.8
>8.46	50.00	21.2-78.8	74.80	66.2-82.2	1.98	0.67	16.2	93.9
>8.48	50.00	21.2-78.8	76.42	67.9-83.6	2.12	0.65	17.1	94.0
>8.51	50.00	21.2-78.8	78.05	69.7-85.0	2.28	0.64	18.2	94.1
>8.52	41.67	15.3-72.2	78.86	70.6-85.7	1.97	0.74	16.1	93.3
>8.56	41.67	15.3-72.2	80.49	72.4-87.1	2.14	0.72	17.2	93.4
>8.57	41.67	15.3-72.2	82.11	74.2-88.4	2.33	0.71	18.5	93.5
>8.58	41.67	15.3-72.2	82.93	75.1-89.1	2.44	0.70	19.2	93.6
>8.59	41.67	15.3-72.2	83.74	76.0-89.8	2.56	0.70	20.0	93.6
>8.6	41.67	15.3-72.2	84.55	76.9-90.4	2.70	0.69	20.8	93.7
>8.61	41.67	15.3-72.2	85.37	77.9-91.1	2.85	0.68	21.7	93.8
>8.63	41.67	15.3-72.2	86.18	78.8-91.7	3.01	0.68	22.7	93.8
>8.66	41.67	15.3-72.2	86.99	79.7-92.4	3.20	0.67	23.8	93.9
>8.67	41.67	15.3-72.2	87.80	80.7-93.0	3.42	0.66	25.0	93.9
>8.69	41.67	15.3-72.2	88.62	81.6-93.6	3.66	0.66	26.3	94.0
>8.7	41.67	15.3-72.2	89.43	82.6-94.2	3.94	0.65	27.8	94.0
>8.71	33.33	10.1-65.1	89.43	82.6-94.2	3.15	0.75	23.5	93.2
>8.73	33.33	10.1-65.1	90.24	83.6-94.9	3.42	0.74	25.0	93.3
>8.74	33.33	10.1-65.1	91.06	84.6-95.4	3.73	0.73	26.7	93.3
>8.75	33.33	10.1-65.1	91.87	85.6-96.0	4.10	0.73	28.6	93.4
>8.79	33.33	10.1-65.1	92.68	86.6-96.6	4.56	0.72	30.8	93.4
>8.81	33.33	10.1-65.1	94.31	88.6-97.7	5.86	0.71	36.4	93.5
>8.84	33.33	10.1-65.1	95.12	89.7-98.2	6.83	0.70	40.0	93.6
>8.85	33.33	10.1-65.1	95.93	90.8-98.7	8.20	0.69	44.4	93.7
>8.87	33.33	10.1-65.1	96.75	91.9-99.1	10.25	0.69	50.0	93.7
>8.93	25.00	5.8-57.2	97.56	93.0-99.5	10.25	0.77	50.0	93.0
>8.94	25.00	5.8-57.2	98.37	94.2-99.8	15.38	0.76	60.0	93.1
>8.97	16.67	2.6-48.4	98.37	94.2-99.8	10.25	0.85	50.0	92.4
>9.04	8.33	1.4-38.5	98.37	94.2-99.8	5.13	0.93	33.3	91.7
>9.08	8.33	1.4-38.5	100.00	97.0-100.0		0.92	100.0	91.8
>10.15	0.00	0.0-26.6	100.00	97.0-100.0		1.00		91.1

+LR: Positive likelihood rati; -LR: Negative likelihood ratio; +PV: Positive predictive value; -PV: Negative predictive value.

**Supplementary Table 4:** Criterion values and coordinates of the ROC curve

Criterion	Sensitivity	95% CI	Specificity	95% CI	+LR	-LR	+PV	-PV
≥4.0101	100.00	73.4-100.0	0.00	0.0-3.0	1.00		8.9	
>4.0101	100.00	73.4-100.0	0.81	0.1-4.5	1.01	0.00	9.0	100.0
>4.0209	100.00	73.4-100.0	1.63	0.2-5.8	1.02	0.00	9.0	100.0
>4.0228	100.00	73.4-100.0	3.25	0.9-8.1	1.03	0.00	9.2	100.0
>4.0531	100.00	73.4-100.0	4.07	1.3-9.2	1.04	0.00	9.2	100.0
>4.0687	100.00	73.4-100.0	4.88	1.8-10.3	1.05	0.00	9.3	100.0
>4.0703	100.00	73.4-100.0	5.69	2.3-11.4	1.06	0.00	9.4	100.0
>4.0842	100.00	73.4-100.0	6.50	2.9-12.4	1.07	0.00	9.4	100.0
>4.0955	100.00	73.4-100.0	7.32	3.4-13.4	1.08	0.00	9.5	100.0
>4.1107	100.00	73.4-100.0	8.13	4.0-14.4	1.09	0.00	9.6	100.0
>4.1247	100.00	73.4-100.0	8.94	4.6-15.4	1.10	0.00	9.7	100.0
>4.1356	100.00	73.4-100.0	9.76	5.1-16.4	1.11	0.00	9.8	100.0
>4.1441	100.00	73.4-100.0	10.57	5.8-17.4	1.12	0.00	9.8	100.0
>4.1506	100.00	73.4-100.0	11.38	6.4-18.4	1.13	0.00	9.9	100.0
>4.1562	100.00	73.4-100.0	12.20	7.0-19.3	1.14	0.00	10.0	100.0
>4.1599	100.00	73.4-100.0	13.01	7.6-20.3	1.15	0.00	10.1	100.0
>4.1628	100.00	73.4-100.0	13.82	8.3-21.2	1.16	0.00	10.2	100.0
>4.1666	100.00	73.4-100.0	14.63	8.9-22.1	1.17	0.00	10.3	100.0
>4.1756	100.00	73.4-100.0	15.45	9.6-23.1	1.18	0.00	10.3	100.0
>4.1832	100.00	73.4-100.0	16.26	10.2-24.0	1.19	0.00	10.4	100.0
>4.1855	100.00	73.4-100.0	17.07	10.9-24.9	1.21	0.00	10.5	100.0
>4.186	100.00	73.4-100.0	17.89	11.6-25.8	1.22	0.00	10.6	100.0
>4.1884	100.00	73.4-100.0	18.70	12.2-26.7	1.23	0.00	10.7	100.0
>4.189	100.00	73.4-100.0	19.51	12.9-27.6	1.24	0.00	10.8	100.0
>4.1956	100.00	73.4-100.0	20.33	13.6-28.5	1.26	0.00	10.9	100.0
>4.1974	100.00	73.4-100.0	21.14	14.3-29.4	1.27	0.00	11.0	100.0
>4.2187	100.00	73.4-100.0	21.95	15.0-30.3	1.28	0.00	11.1	100.0
>4.2355	100.00	73.4-100.0	22.76	15.7-31.2	1.29	0.00	11.2	100.0
>4.236	100.00	73.4-100.0	23.58	16.4-32.1	1.31	0.00	11.3	100.0
>4.2382	100.00	73.4-100.0	24.39	17.1-33.0	1.32	0.00	11.4	100.0
>4.2421	100.00	73.4-100.0	25.20	17.8-33.8	1.34	0.00	11.5	100.0
>4.2435	100.00	73.4-100.0	26.83	19.2-35.6	1.37	0.00	11.8	100.0
>4.2456	100.00	73.4-100.0	27.64	20.0-36.4	1.38	0.00	11.9	100.0
>4.248	100.00	73.4-100.0	28.46	20.7-37.3	1.40	0.00	12.0	100.0
>4.2536	100.00	73.4-100.0	29.27	21.4-38.2	1.41	0.00	12.1	100.0
>4.2546	100.00	73.4-100.0	30.08	22.1-39.0	1.43	0.00	12.2	100.0
>4.2578	100.00	73.4-100.0	30.89	22.9-39.9	1.45	0.00	12.4	100.0
>4.2681	100.00	73.4-100.0	31.71	23.6-40.7	1.46	0.00	12.5	100.0
>4.2801	100.00	73.4-100.0	32.52	24.4-41.6	1.48	0.00	12.6	100.0
>4.2844	100.00	73.4-100.0	33.33	25.1-42.4	1.50	0.00	12.8	100.0
>4.2877	100.00	73.4-100.0	34.15	25.8-43.2	1.52	0.00	12.9	100.0
>4.2896	100.00	73.4-100.0	34.96	26.6-44.1	1.54	0.00	13.0	100.0
>4.2942	100.00	73.4-100.0	35.77	27.3-44.9	1.56	0.00	13.2	100.0
>4.3026	100.00	73.4-100.0	36.59	28.1-45.7	1.58	0.00	13.3	100.0
>4.3085	100.00	73.4-100.0	37.40	28.8-46.6	1.60	0.00	13.5	100.0
>4.3102	100.00	73.4-100.0	38.21	29.6-47.4	1.62	0.00	13.6	100.0
>4.314	100.00	73.4-100.0	39.02	30.4-48.2	1.64	0.00	13.8	100.0
>4.3167	100.00	73.4-100.0	39.84	31.1-49.1	1.66	0.00	14.0	100.0
>4.3259	100.00	73.4-100.0	40.65	31.9-49.9	1.68	0.00	14.1	100.0
>4.3352	100.00	73.4-100.0	41.46	32.7-50.7	1.71	0.00	14.3	100.0
>4.341	100.00	73.4-100.0	42.28	33.4-51.5	1.73	0.00	14.5	100.0
>4.343	100.00	73.4-100.0	43.09	34.2-52.3	1.76	0.00	14.6	100.0
>4.3437	100.00	73.4-100.0	43.90	35.0-53.1	1.78	0.00	14.8	100.0
>4.3447	100.00	73.4-100.0	44.72	35.7-53.9	1.81	0.00	15.0	100.0
>4.356	100.00	73.4-100.0	45.53	36.5-54.8	1.84	0.00	15.2	100.0
>4.3561	100.00	73.4-100.0	46.34	37.3-55.6	1.86	0.00	15.4	100.0
>4.3566	100.00	73.4-100.0	47.15	38.1-56.4	1.89	0.00	15.6	100.0
>4.3742	100.00	73.4-100.0	47.97	38.9-57.2	1.92	0.00	15.8	100.0
>4.377	100.00	73.4-100.0	48.78	39.7-58.0	1.95	0.00	16.0	100.0
>4.3797	100.00	73.4-100.0	49.59	40.5-58.7	1.98	0.00	16.2	100.0
>4.3801	100.00	73.4-100.0	50.41	41.3-59.5	2.02	0.00	16.4	100.0
>4.3882 *	100.00	73.4-100.0	51.22	42.0-60.3	2.05	0.00	16.7	100.0
>4.3938	91.67	61.5-98.6	51.22	42.0-60.3	1.88	0.16	15.5	98.4
>4.3941	91.67	61.5-98.6	52.03	42.8-61.1	1.91	0.16	15.7	98.5
>4.3951	91.67	61.5-98.6	52.85	43.6-61.9	1.94	0.16	15.9	98.5
>4.3964	91.67	61.5-98.6	53.66	44.4-62.7	1.98	0.16	16.2	98.5
>4.4083	91.67	61.5-98.6	54.47	45.2-63.5	2.01	0.15	16.4	98.5
>4.4167	83.33	51.6-97.4	55.28	46.1-64.3	1.86	0.30	15.4	97.1
>4.4237	83.33	51.6-97.4	56.10	46.9-65.0	1.90	0.30	15.6	97.2
>4.4307	83.33	51.6-97.4	56.91	47.7-65.8	1.93	0.29	15.9	97.2
>4.4367	75.00	42.8-94.2	56.91	47.7-65.8	1.74	0.44	14.5	95.9
>4.437	75.00	42.8-94.2	57.72	48.5-66.6	1.77	0.43	14.8	95.9
>4.4424	75.00	42.8-94.2	58.54	49.3-67.3	1.81	0.43	15.0	96.0
>4.4445	66.67	34.9-89.9	58.54	49.3-67.3	1.61	0.57	13.6	94.7
>4.4512	66.67	34.9-89.9	59.35	50.1-68.1	1.64	0.56	13.8	94.8
>4.4546	66.67	34.9-89.9	60.16	50.9-68.9	1.67	0.55	14.0	94.9
>4.4708	66.67	34.9-89.9	60.98	51.8-69.6	1.71	0.55	14.3	94.9
>4.4763	66.67	34.9-89.9	61.79	52.6-70.4	1.74	0.54	14.5	95.0
>4.4826	66.67	34.9-89.9	62.60	53.4-71.2	1.78	0.53	14.8	95.1
>4.4864	66.67	34.9-89.9	63.41	54.3-71.9	1.82	0.53	15.1	95.1
>4.4893	66.67	34.9-89.9	64.23	55.1-72.7	1.86	0.52	15.4	95.2
>4.4936	66.67	34.9-89.9	65.04	55.9-73.4	1.91	0.51	15.7	95.2
>4.4994	58.33	27.8-84.7	65.04	55.9-73.4	1.67	0.64	14.0	94.1
>4.5046	58.33	27.8-84.7	65.85	56.8-74.2	1.71	0.63	14.3	94.2
>4.5146	58.33	27.8-84.7	66.67	57.6-74.9	1.75	0.62	14.6	94.3
>4.518	58.33	27.8-84.7	67.48	58.4-75.6	1.79	0.62	14.9	94.3
>4.5256	58.33	27.8-84.7	68.29	59.3-76.4	1.84	0.61	15.2	94.4
>4.5378	58.33	27.8-84.7	69.11	60.1-77.1	1.89	0.60	15.6	94.4
>4.5444	58.33	27.8-84.7	69.92	61.0-77.9	1.94	0.60	15.9	94.5
>4.5462	50.00	21.2-78.8	69.92	61.0-77.9	1.66	0.72	14.0	93.5
>4.5485	50.00	21.2-78.8	71.54	62.7-79.3	1.76	0.70	14.6	93.6
>4.5508	50.00	21.2-78.8	72.36	63.6-80.0	1.81	0.69	15.0	93.7
>4.563	50.00	21.2-78.8	73.17	64.4-80.8	1.86	0.68	15.4	93.7
>4.5721	50.00	21.2-78.8	73.98	65.3-81.5	1.92	0.68	15.8	93.8
>4.5747	50.00	21.2-78.8	74.80	66.2-82.2	1.98	0.67	16.2	93.9
>4.5845	50.00	21.2-78.8	75.61	67.0-82.9	2.05	0.66	16.7	93.9
>4.5868	50.00	21.2-78.8	76.42	67.9-83.6	2.12	0.65	17.1	94.0
>4.6008	50.00	21.2-78.8	77.24	68.8-84.3	2.20	0.65	17.6	94.1
>4.603	50.00	21.2-78.8	78.05	69.7-85.0	2.28	0.64	18.2	94.1
>4.6068	41.67	15.3-72.2	78.05	69.7-85.0	1.90	0.75	15.6	93.2
>4.6081	41.67	15.3-72.2	78.86	70.6-85.7	1.97	0.74	16.1	93.3
>4.6271	41.67	15.3-72.2	79.67	71.5-86.4	2.05	0.73	16.7	93.3
>4.6284	41.67	15.3-72.2	80.49	72.4-87.1	2.14	0.72	17.2	93.4
>4.6297	41.67	15.3-72.2	81.30	73.3-87.8	2.23	0.72	17.9	93.5
>4.6315	41.67	15.3-72.2	82.11	74.2-88.4	2.33	0.71	18.5	93.5
>4.6386	41.67	15.3-72.2	82.93	75.1-89.1	2.44	0.70	19.2	93.6
>4.6395	41.67	15.3-72.2	83.74	76.0-89.8	2.56	0.70	20.0	93.6
>4.6444	41.67	15.3-72.2	84.55	76.9-90.4	2.70	0.69	20.8	93.7
>4.6537	41.67	15.3-72.2	85.37	77.9-91.1	2.85	0.68	21.7	93.8
>4.6615	41.67	15.3-72.2	86.18	78.8-91.7	3.01	0.68	22.7	93.8
>4.6744	41.67	15.3-72.2	86.99	79.7-92.4	3.20	0.67	23.8	93.9
>4.6814	41.67	15.3-72.2	87.80	80.7-93.0	3.42	0.66	25.0	93.9
>4.6898	41.67	15.3-72.2	88.62	81.6-93.6	3.66	0.66	26.3	94.0
>4.6968	41.67	15.3-72.2	89.43	82.6-94.2	3.94	0.65	27.8	94.0
>4.703	33.33	10.1-65.1	89.43	82.6-94.2	3.15	0.75	23.5	93.2
>4.7128	33.33	10.1-65.1	90.24	83.6-94.9	3.42	0.74	25.0	93.3
>4.7157	33.33	10.1-65.1	91.06	84.6-95.4	3.73	0.73	26.7	93.3
>4.7197	33.33	10.1-65.1	91.87	85.6-96.0	4.10	0.73	28.6	93.4
>4.74	33.33	10.1-65.1	92.68	86.6-96.6	4.56	0.72	30.8	93.4
>4.7494	33.33	10.1-65.1	93.50	87.6-97.1	5.12	0.71	33.3	93.5
>4.753	33.33	10.1-65.1	94.31	88.6-97.7	5.86	0.71	36.4	93.5
>4.7651	33.33	10.1-65.1	95.12	89.7-98.2	6.83	0.70	40.0	93.6
>4.771	33.33	10.1-65.1	95.93	90.8-98.7	8.20	0.69	44.4	93.7
>4.7812	33.33	10.1-65.1	96.75	91.9-99.1	10.25	0.69	50.0	93.7
>4.8114	25.00	5.8-57.2	96.75	91.9-99.1	7.69	0.78	42.9	93.0
>4.8122	25.00	5.8-57.2	97.56	93.0-99.5	10.25	0.77	50.0	93.0
>4.8143	25.00	5.8-57.2	98.37	94.2-99.8	15.38	0.76	60.0	93.1
>4.8294	16.67	2.6-48.4	98.37	94.2-99.8	10.25	0.85	50.0	92.4
>4.8669	8.33	1.4-38.5	98.37	94.2-99.8	5.13	0.93	33.3	91.7
>4.8885	8.33	1.4-38.5	99.19	95.5-99.9	10.25	0.92	50.0	91.7
>4.8887	8.33	1.4-38.5	100.00	97.0-100.0		0.92	100.0	91.8
>5.4228	0.00	0.0-26.6	100.00	97.0-100.0		1.00		91.1

+LR: Positive likelihood ratio; -LR: Negative likelihood ratio; +PV: Positive predictive value; -PV: Negative predictive value.

**STROBE Statement:** a checklist of items that should be included in reports of observational studies

	Item No.	Recommendation	Page No.
**Title and abstract**	1	(*a*) Indicate the study’s design with a commonly used term in the title or the abstract	1-2
		(*b*) Provide in the abstract an informative and balanced summary of what was done and what was found	2
**Introduction**			
Background/rationale	2	Explain the scientific background and rationale for the investigation being reported	3-4
Objectives	3	State specific objectives, including any prespecified hypotheses	4
**Methods**			
Study design	4	Present key elements of study design early in the paper	4
Setting	5	Describe the setting, locations, and relevant dates, including periods of recruitment, exposure, follow-up, and data collection	4-5
Participants	6	(*a*) *Cross-sectional study* – Give the eligibility criteria and the sources and methods of selection of participants	4-5
	
Variables	7	Clearly define all outcomes, exposures, predictors, potential confounders, and effect modifiers. Give diagnostic criteria, if applicable	5-8
Data sources/ measurement	8*	For each variable of interest, give sources of data and details of assessment methods (measurement). Describe comparability of assessment methods if there is more than one group	5-8
Bias	9	Describe any efforts to address potential sources of bias	6-7
Study size	10	Explain how the study size was arrived at	5
Quantitative variables	11	Explain how quantitative variables were handled in the analyses. If applicable, describe which groupings were chosen and why	8
Statistical methods	12	(*a*) Describe all statistical methods, including those used to control for confounding	8
		(*b*) Describe any methods used to examine subgroups and interactions	8
		(*c*) Explain how missing data were addressed	8
		(*d*) *Cohort study* – If applicable, explain how loss to follow-up was addressed *Case-control study* – If applicable, explain how matching of cases and controls was addressed *Cross-sectional study* – If applicable, describe analytical methods taking account of sampling strategy	-
		(*e*) Describe any sensitivity analyses	
Participants	13*	(a) Report numbers of individuals at each stage of study – e.g., numbers potentially eligible, examined for eligibility, confirmed eligible, included in the study, completing follow-up, and analyzed	5
		(b) Give reasons for non-participation at each stage	5
		(c) Consider use of a flow diagram	5 (Figure 1)
Descriptive data	14*	(a) Give characteristics of study participants (*e.g.* demographic, clinical, social) and information on exposures and potential confounders	8-9
		(b) Indicate number of participants with missing data for each variable of interest	5
		(c) *Cohort study* – Summarize follow-up time (*e.g.*, average and total amount)	
Outcome data	15*	*Cohort study* – Report numbers of outcome events or summary measures over time	
		*Case-control study* – Report numbers in each exposure category, or summary measures of exposure	
		*Cross-sectional study* – Report numbers of outcome events or summary measures	8-9
Main results	16	(*a*) Give unadjusted estimates and, if applicable, confounder-ººadjusted estimates and their precision (*e.g.*, 95% confidence interval). Make clear which confounders were adjusted for and why they were included	8-9
		(*b*) Report category boundaries when continuous variables were categorized	8-9
		(*c*) If relevant, consider translating estimates of relative risk into absolute risk for a meaningful time period	9
Other analyses	17	Report other analyses done –*e.g.* analyses of subgroups and interactions, and sensitivity analyses	-
Key results	18	Summarize key results with reference to study objectives	9-10
Limitations	19	Discuss limitations of the study, taking into account sources of potential bias or imprecision. Discuss both direction and magnitude of any potential bias	13-14
Interpretation	20	Give a cautious overall interpretation of results considering objectives, limitations, multiplicity of analyses, results from similar studies, and other relevant evidence	10-13
Generalizability	21	Discuss the generalizability (external validity) of the study results	13
Other information			
Funding	22	Give the source of funding and the role of the funders for the present study and, if applicable, for the original study on which the present article is based	14

* Give information separately for cases and controls in case-control studies and, if applicable, for exposed and unexposed groups in cohort and cross-sectional studies.

Note: An Explanation and Elaboration article discusses each checklist item and gives methodological background and published examples of transparent reporting. The STROBE checklist is best used in conjunction with this article (freely available on the Web sites of PLoS Medicine at http://www.plosmedicine.org/, Annals of Internal Medicine at http://www.annals.org/, and Epidemiology at http://www.epidem.com/). Information on the STROBE Initiative is available at www.strobe-statement.org.
